# Ecological and Human Health Risks of Heavy Metals in Shooting Range Soils: A Meta Assessment from China

**DOI:** 10.3390/toxics8020032

**Published:** 2020-05-01

**Authors:** Juan Bai, Xiaofen Zhao

**Affiliations:** 1 College of Physical Education, Qingdao University of Science and Technology, Qingdao 266061, China; baijuan@qust.edu.cn; 2 Library, Qingdao University of Science and Technology, Qingdao 266061, China

**Keywords:** health risk, heavy metals, shooting range, ecological risk, soil pollution

## Abstract

Contamination of shooting ranges by heavy metals in particular Pb represents a widespread environmental issue attracting concern worldwide. Contaminant accumulation in shooting range soils can pose potential ecological risks and health risks for shooters and workers. Based on the published data on metal contamination at five shooting ranges in China, potential ecological and human health risks of several metals, and in particular, Pb were assessed for the five surveyed shooting ranges. Data show the mean concentrations of Pb, Cu, Hg, Sb, Ni and Cr in various ranges were all higher than the local soil background values, implying their accumulation was induced by shooting activities. The degree of contamination varied with sites and metals, very high Pb contamination at Range 1, Range 2 and Range 5-1, while moderate Pb contamination at Range 3 and Range 5-2. Comparatively, As, Zn and Co showed no contamination. Among the surveyed metals, Pb, Cu, Hg and Sb in shooting range soils displayed relatively high potential ecological risks. The overall degree of potential ecological risk was very high at Range 1 and Range 2, considerable at Range 4 and Range 5-1, and low at Range 3 and Range 5-2. The mean HI (hazard index) of Pb at Range 2 and the maximum HI values at Range 1 and Range 4 were higher than 1, suggesting a possibility of non-carcinogenic risks of Pb contamination at these sites. However, Pb in other range soils and other metals, across the five ranges, all exhibited no non-carcinogenic risks. The cancer risks of the four carcinogenic contaminants (As, Co, Cr, and Ni) were acceptable or negligible at all ranges. In conclusion, contamination of Pb and other metals such as Cu, Hg and Sb can cause various potential ecological risks at all the surveyed ranges, but only Pb at three ranges shows possible health risks. Contamination of Pb in the surveyed shooting ranges should be managed to reduce its possible environmental and health risks.

## 1. Introduction

Toxic metals without known biological functions (e.g., Pb, Cd, and As) and even some essential metals for human beings (e.g., Cu, Zn, and Cr) can cause health risks when present in excess levels. It is well known that excessive intake of Pb can cause damages in human biological systems such as nervous system, immune system and reproductive system, and especially children’s brain development [1]. Some toxic metals, such as Pb, As, Cd and Cr have been classified as carcinogenic elements by the International Agency for Research on Cancer [2]. Thus, contaminant status and potential risks of the sites, closely related to human health, require serious consideration.

Due to the composition of metals in bullets and shells, shooting activity has been recognized as an important soil pollution source of heavy metals, such as Pb [3,4]. It is estimated that 10 to 60,000 tons of Pb is released annually in shooting ranges in different countries, ranked as the second largest source of Pb contamination after battery industry [5]. According to an estimate by U.S. Environmental Protection Agency (USEPA), about 7.26 × 10^4^ ton of Pb shot and bullets are used annually at approximately 9000 nonmilitary shooting ranges [6]. The discharged shot and bullets, as well as the release of Pb powder, lead to Pb accumulation in shooting range soils [4]. Numerous investigations have confirmed that Pb can accumulate in soil at and around shooting ranges after years of shooting activity, and ultimately reach pollution levels. For instance, Pb concentrations of 10,000–70,000 mg/kg have been reported in shooting range soil [7]. At another shooting range in the US, Pb concentrations varied ranging from 27,000 to 233,142 mg/kg [8]. Furthermore, due to the complex components of bullets/shots used in shooting, contaminants such as Cu, Sb, Zn and Ni were also reported in shooting range soils [3]. A case study from small arms shooting ranges showed that the concentrations of Pb, Cu, Zn and Sb reached 13, 5.2, 1.1, and 0.83 g/kg, respectively [9]. Undoubtedly, soil contamination with Pb, as well as the co-contaminants may cause direct and indirect consequences for human health.

As summarized in two reviews [3,4], previous studies were focused mainly on the contamination status, environmental fate and management of Pb and other co-contaminants in shooting range soils. Particularly, Pb in shooting range soils can be highly bioavailable, indicating a potential toxicity and environmental risk [3]. However, only few studies assessed their environmental and health risks [10,11,12,13]. Although several studies investigated heavy metal contamination status of shooting ranges in China, to our knowledge, no studies have been conducted on their ecological and health risks. In our present study, we assessed the potential ecological risks and health risks of the contaminants, based on the published data on heavy metal contamination in shooting ranges in China.

## 2. Materials and Methods

### 2.1. Data Collecting

We searched the published journal and conference papers based on several international databases, such as Google Scholar, Scopus, Web of Science, and the biggest database in China (China National Knowledge Infrastructure, CNKI). The following search items were included: Shooting range contamination/pollution, shooting range soil, lead/heavy metals, and/or China. Finally, five papers published in international and Chinese journals were collected (see Table 1). In total, five shooting ranges were studied, among which, the fifth was divided into two sampling areas for investigation. Thus, the ranges were numbered in chronological order as Range 1, Rang 2, Range 3, Range 4, Rang 5-1, and Range 5-2, respectively.

Thereafter, the data on heavy metal concentrations in tables and figures in the published papers were extracted directly, or using the software GetData Graph Digitizer version 2.26 (GETDATA Graph Digitizer, Moscow, Russia, 2013). The mean, maximum and minimum values were used for calculation of risk assessment.

### 2.2. Assessment of Contamination Degree and Potential Ecological Risk

The following equations were used to assess the contamination degree and potential ecological risk of heavy metals in shooting range soils [14]:

(1) $$\mathrm{CF}=\frac{C_{\mathrm{metal}}}{C_{\mathrm{background}}}$$

(2) $$\mathrm{CD}=\sum_{i=1}^{n}\mathrm{CF}$$

Eri = Tr × CF(3)

(4) $$\mathrm{RI}=\sum_{i=1}^{n}\mathrm{Eri}.$$

Contamination factor (CF) can be calculated using the ratio of the specific metal concentration in soil to its soil background value (Equation (1)). The concentration of each individual metal (C_metal_) was from the published data (Table 1). The background values (C_background_) of these metals were shown in Table S1 [15]. The degree of contamination (CD) is the summation of the CF of each metal (Equation (2)). The toxic response factor (Tr) for the given metal was cited from previous references [14,16,17] (Table S1). The ecological risk factor (Eri) is defined as the product of CF and Tr (Equation (3)), and ecological risk index (RI) is defined as the sum of Eri of all metals in soil (Equation (4)). The classification of degree of contamination and ecological risk is shown in Table S2 [14].

### 2.3. Health Risk Assessment

Health risks of contaminants were evaluated based on USEPA’s Exposure Factors Handbook and Exposure Factors Handbook of Chinese Population (Adults) [18]. Average daily doses (ADDs) of contaminants via ingestion, inhalation and dermal contact were calculated based on the following Equations (Equations (5)–(7)). The parameters in the Equations (5)–(7) were presented in Table S3:

(5) $${\mathrm{ADD}}_{\mathrm{ing}}\;=C_{\mathrm{metal}}\;\times \;\frac{\mathrm{IngR}\times \mathrm{TEF}\times \mathrm{ED}}{\mathrm{BW}\times \mathrm{AT}}\;\times \;10^{-6}$$

(6) $${\mathrm{ADD}}_{\mathrm{inh}}\;=C_{\mathrm{metal}}\;\times \;\frac{\mathrm{InhR}\times \mathrm{TEF}\times \mathrm{ED}}{\mathrm{PEF}\times \mathrm{BW}\times \mathrm{AT}}$$

(7) $${\mathrm{ADD}}_{\mathrm{der}}\;=C_{\mathrm{metal}}\;\times \;\frac{\mathrm{SL}\times \mathrm{SA}\times \mathrm{ABS}\times \mathrm{TEF}\times \mathrm{ED}}{\mathrm{BW}\times \mathrm{AT}}\;\times \;10^{-6}$$

The potential non-carcinogenic and carcinogenic risks were calculated using hazard quotient (HQ), and cancer risk (CR), respectively (Equations (8) and (9)). Hazard index (HI) is the summation of the HQs for the three exposure routes (Equation (10)). The value of HQ or HI > 1 means a possibility of non-carcinogenic risk, and the value of HQ or HI < 1 means no significant non-carcinogenic risk. The total carcinogenic risk (TR) is the summation of the CRs for the three exposure routes (Equation (11)). The cancer risk is negligible when CR or TR lower than 1 × 10^−6^, and acceptable or tolerable for the range of 1 × 10^−6^ to 1 × 10^−4^. The value of CR or TR higher than 1 × 10^−4^ means that 1 in 10,000 persons may develop cancer from lifetime exposure to carcinogenic risks. The definitions and values of RfD and SF were presented in Table S4 [16,19,20,21]:

(8) $$\mathrm{HQ}=\frac{\mathrm{ADD}}{\mathrm{RfD}}$$

(9) HI=∑HQ

CR = ADD × SF(10)

(11) TR=∑CR.

### 2.4. Data Analysis

The mean, maximum and minimum concentrations of each metal were used for calculating the above parameters using Excel 2016 (Microsoft Corporation, Redmond, WA, USA).

## 3. Results and Discussion

### 3.1. Heavy Metal Concentrations in Shooting Range Soils of China

A total of five shooting ranges were listed in chronological order (Table 1). These shooting ranges (except Range 1) were known to have been open for more than 15 years. Among them, only Range 3 was tank shooting range, while others were all involved in small arms, such as pistols, rifles, and submachine guns.

Generally, high Pb concentrations were detected in soils of small arms shooting ranges (Range 1, Range 2, Range 4, and Range 5-1), with the highest value of 7674 mg/kg at Range 2. However, large variations were observed, and the maximum concentrations were one to two orders of magnitude higher than the minimum values. Comparatively, in the tank shooting range (Range 3), Pb concentrations were much lower. Even in the same range, Pb concentrations were much lower in non-bomb dropping area than in bomb-beaten area. In addition to Pb, some other metals including Cu, Hg, As, Sb, Zn, Ni, Cr and Co were also detected in some ranges.

Obviously, the accumulation of metals resulted mainly from gunshot residues released by shooting activities, such as fragments of the bullet, cartridge case, burnt and unburnt particles from gunpowder. Ammunitions of light and heavy weapons often contained heavy metals. The most common bullets consist of a Pb core and a Cu outer jacket. It is estimated that most bullets contain over 90% Pb, 1 to 5% Sb, and up to 9% Cu for jacketed bullets, and some other minor alloying agents such as Zn, As, and Ni [6]. In Pb-bullets, 95–97% of the weight is metallic Pb, following by 0.4–2.0% Sb, 0.2–0.8% As, and other metals such as Sn, Se, Mn, Cd, Cr, Cu, and Ni [28]. Hg compounds were used in primers for rifle and handgun ammunition [29]. In tank shells, the common components are steel alloy, brass, Cu/Zn alloy, Ni alloy and aluminum alloy [24]. After the shooting activities, the abrasion and weathering of fired bullets or shots lead to metal release and accumulation in shooting range soils.

According to the “Soil Environmental Quality–Risk control standard for soil contamination of agricultural land (GB 15618-2018)”, most Pb concentrations in small arms shooting ranges exceed this risk screening value, indicating risk assessment should be performed for these sites. Maximum values of Pb in some sites and maximum Hg value in Range 1 exceed their risk intervention values, indicating risk control and remediation measures should be taken for these sites.

### 3.2. Assessment of Contamination Degree

As shown in Table 2, the mean CF values of Pb, Cu, Hg, Sb, Ni and Cr were all higher than 1, while the values of As, Zn and Co were lower than 1. Pb contamination degree varied with sites, with very high contamination in Range 1, Range 2 and Range 5-1, while moderate contamination in Range 3 and Range 5-2. Cu contamination degree reached very high in Range 4 and Range 5-1, considerable in Range 1, and moderate in Range 3 and Range 5-2. The mean CF value of Hg in Range 1 was as high as 567.7, representing a very high degree of contamination. The mean CF values of Sb, Ni and Cr all ranged from 1 to 3, indicating moderate contamination at these sites. As, Zn and Co levels at the surveyed sites were classified as low contamination. The CD values confirmed that very high contamination occurred in Range 1, Range 2, Range 4, and Range 5-1. However, the low contamination degree in Range 3 may not reflect the real situation, because not all metals were fully investigated at this site, particularly the metals with low background values (e.g., Hg and Sb).

The present data confirm heavy metal contamination particularly Pb contamination at all the surveyed shooting ranges, but the contamination degree varies greatly with sampling location and shooting range type. Due to the variability of shooting activities, metal pollution is unlikely to occur evenly in the range. Pb contamination generally shows heterogeneity at shooting ranges, with the highest density of Pb bullets usually in the berm [4]. For pistol and rifle ranges, high concentrations of contaminants, Pb in particular, generally accumulate in bullet berms and shot fall zones [3]. The highest total Pb concentration of 38406 mg/kg was detected in the berm soils of Thebephatshwa shooting range [30]. For target ranges, a maximum value of 26,700 mg/kg Pb was observed near the center of the expected shot fallout zone [31]. Thus, it is probable that bomb-beaten zone had higher Pb concentrations than non-bomb dropping area at Range 3.

On the other hand, metal species and concentrations in shooting range soil depend on the components of bullets or shells, while metallic components of bullets or shots vary by purpose and by manufacturer [6]. Most common bullets contain over 90% Pb, as well as other minor components such as Cu, Ab, As, Cr, Ni [6,28]. In contrast, in tank shells, the common components are steel alloy, brass, copper-zinc alloy, and other alloys [24], which can explain the reason high levels of Pb generally accumulated in the small arms shooting ranges, but not in the tank shooting range. Meanwhile, the components of minor metals in bullets and shells can also explain their accumulation in soil. Thus, it can be concluded that, heavy metal concentrations and contamination degrees in shooting ranges are site-specific, varying greatly with the type and sampling location.

### 3.3. Assessment of Potential Ecological Risk

Based on Eri values shown in Table 2, potential ecological risk of Pb could be classified as very high for Range 2 and Range 4, high for Range 1 and Range 5-1, and low for Range 3 and Range 5-2. The mean Eri values of Cu at Range 4 and Range 5-1, and the maximum Eri values of Cu at Range 1 and Range 5-2 were all higher than 40, but lower than 160, indicating moderate or considerable potential ecological risks of Cu at these sites. For Range 1, even the minimum Eri value of Hg reached as high as 8430.8, meaning its very high potential ecological risk. Although the mean Eri values of Sb at Range 1 did not exceed 40, but the maximum value reached as high as 115.7, which means at least some sampling points of Range 1 have considerable potential ecological risk. For other metals Zn, Cr, Ni and Co, all their Eri values were lower than 40, suggesting their low risks at these sites. Considering the values of IR, the overall degree of potential ecological risk was very high at Range 1 and Range 2, considerable at Range 4 and Range 5-1, and low at Range 3 and Range 5-2.

The present data confirm potential ecological risks of Pb, Cu, Hg and Sb in shooting range soils. Generally, Pb is considered the most widespread risky contaminant in most shooting ranges. At two military shooting ranges in Korea, Eri value of Pb reached 3446, and 725, respectively [12]. In seven shooting ranges in Botswana, the Eri value of Pb ranged from 1192 to 56,272 [13]. Peddicord and LaKind [11] first evaluated the ecological risks of shooting rang contaminants for some animals, and found that Pb posed substantial risks to small mammals and grit-ingesting birds, but other contaminants, including As, Sb, Cu, Zn and PAHs posed minimal risks. One rifle and shotgun shooting site in Finland was shown to have high ecological risks to terrestrial and aquatic organisms, and Pb was the most important contaminant. However, the data from Range 1 showed that Hg had much higher ecological risks than Pb on Range 1. Since Hg can persist in shooting rang soil for a long term [29], Hg contamination and environmental risks deserves more attention in future study. Furthermore, considering their potential ecological risks, contamination of Cu and Sb at shooting ranges are also not negligible.

More interestingly, contaminants in shooting range soils have been confirmed to accumulate in organisms inhabiting there. Pb concentration in *Stipa krylovii* plants grown in the soil at Range 1 was 16 times of that in the control plants [22]. In another study, Pb at rifle and pistol ranges could be accumulated by earthworms and vegetation, and imposed elevated risk for their consumers through the food chain [32]. Bioaccumulation or biomagnification of Pb and Cu in plants and invertebrates was observed at shooting sites [33]. Moreover, Pb contamination of an old shooting range, not only negatively affected soil microbes and enchytraeid worms, but also disturbed ecological processes, such as decomposition and nutrient mineralization [34]. Undoubtedly, the ecological risks of shooting ranges at the ecosystem scale need to be assessed.

### 3.4. Health Risk Assessment

The values of HI and TR via the three exposure routes were shown in Table 3. The mean HI value of Pb at Range 2, and the maximum HI values of Pb at Range 1 and Range 4, were higher than 1, suggesting a possibility of non-carcinogenic effects of Pb contamination at these sites. However, Pb in other range soils and other metals across the five ranges all showed no non-carcinogenic risks.

The cancer risks of the four carcinogenic contaminants (As, Co, Cr, and Ni) were measured (Table 4). Generally, TR values of As at Range 1 were higher than 1 × 10^−6^, but lower than 1 × 10^−5^, suggesting that the possible carcinogenic risks from exposure of As were acceptable. However, TR values of Cr, Co and Ni, and their sums were all much lower than 1 × 10^−6^, indicating the potential cancer risks can be considered negligible at Range 3 and Range 4.

Heavy metal accumulation in shooting range soil can pose health risks via exposure routes such as ingestion, respiratory inhalation, and dermal contact, particularly for workers and shooters of shooting ranges [4]. Here, our results indicated that, among the surveyed metals, Pb had the highest possibility of non-carcinogenic risks, and ingestion exposure route accounted for the highest contribution, followed by dermal contact and then inhalation (data not shown). Soil particles or dust-bearing Pb can be released during shooting activities by shooters and maintenance operations by workers, and then produce health risks via exposure routes, such as hand-mouth intake. In particular, Pb dust is not only in direct contact with the workers’ skins, but also adheres to their clothing and shoes, thereby potentially exposing their families to Pb if the dust is taken home.

Although Hg sometimes showed a very high contamination degree and potential ecological risks, its non-carcinogenic risks were negligible. The high ecological risks of Hg are mainly from its high toxicity factor, but its absolute contents are quite low compared to Pb, and thereby human exposure risks are also relatively low. Thus, the contamination degree and ecological risk of Hg, based on the methods in our study, may be overestimated. Other metals, irrespective of their contamination degree and potential ecological risks, all did not show significant carcinogenic and non-carcinogenic risks.

Notably, compared to other contaminants, Pb in shooting ranges is sometimes of high mobility and availability, and may cause contamination of surface and groundwater, thereby imposing risks to the general public [3,4]. Via surface runoff and atmospheric precipitation, contaminants in polluted shooting ranges can spread to surrounding environments and induce adverse impacts on the organisms therein. Therefore, for shooting ranges with potential ecological and health risks, it is necessary to adopt management practices for risk control and environmental remediation.

## 4. Conclusions

Our present results confirm that soil contamination by heavy metals in particular Pb commonly occurred in China’s shooting ranges. Among the surveyed metals, Pb, Cu, Hg and Sb in shooting range soils displayed relatively high potential ecological risks. Due to its high concentration, non-carcinogenic risks of Pb contamination were observed at three of the surveyed sites. However, other metals across the five ranges did not display non-carcinogenic risks. The carcinogenic risks of the four carcinogenic metals (As, Co, Cr, and Ni) were acceptable or negligible at all ranges. In conclusion, contamination of Pb and co-contaminants Cu, Hg and Sb may produce potential ecological risks, but only Pb displays potential health risks. It is necessary to take measures to reduce the environmental and health risks of Pb contamination in shooting range soils.

## Figures and Tables

**Table 1:** Concentrations of various metals in shooting rang soils reported in China.

Number of Ranges	Shooting Range	Value (mg/kg)	Pb	Cu	Hg	As	Sb	Zn	Ni	Cr	Co	References
Range 1	Small arms shooting range: bullet berms and target zones (0–5 cm); pH 7.70–8.65	Mean	936.4	88.4	36.9	9.54	1.97	40.2				[22]
		Max	7031	448	149	13.4	14.0	91.5				
		Min	12.5	11.6	13.7	4.67	0.17	20.3				
Range 2	Small arms shooting range (15 years): bullet berms and target zones (0–5 cm); pH 8.2–9.0	Mean	4175									[23]
		Max	7674									
		Min	132									
Range 3	Tank shooting range (20 years): shot fall and non-shot fall zones (0–15 cm); mean pH 5.96;	Mean	30.33	46.29				56.71	32.73		9.82	[24]
		Max	52.49	101.90				91.27	56.50		19.77	
		Min	20.52	27.25				32.17	19.05		5.03	
Range 4	Small arms shooting range (15 years): shooting and target zones (0–20 cm); mean pH 7.31	Mean	2044.5	183.4				68.1	34.2	88.9		[25]
		Max	5010.0	396.5				91.1	38.8	98.2		
		Min	151.9	44.1				47.9	28.1	80.6		
Range 5-1	Small arms shooting range (20 years): Bomb-beaten area (0–20 cm); mean pH 6.64	Mean	1375.35	214.62								[26]
		Max	2763	306.90								
		Min	414	104								
Range 5-2	Small arms shooting range (20 years): Non-bomb dropping area (0–20 cm); mean pH 6.64	Mean	55.21	41.95								
		Max	153.70	226.20								
		Min	22.58	17.04								
China’s soil environmental quality	Risk screening value		800	18,000	38	60			900			[27]
	Risk intervention value		2500	36,000	82	140			2000			[27]

Note: Blank space means data were not provided. In references [22,23], the metals were determined using an inductively coupled plasma optical emission spectrometer and hydride generation-atomic fluorescence spectrophotometer after microwave digestion. In references [23,24,25], the metals were determined using an inductively coupled plasma optical emission spectrometer after digestion based on USEPA Method 3050B.

**Table 2:** Contamination factor (CF), contamination degree (CD), ecological risk factor (Eri), and potential ecological risk index (RI) of heavy metals in the shooting ranges.

Metal	Value	Range 1			Range 2			Range 3			Range 4			Range 5-1			Range 5-2		
		Mean	Max	Min	Mean	Max	Min	Mean	Max	Min	Mean	Max	Min	Mean	Max	Min	Mean	Max	Min
Pb	CF	36.0	270.4	0.5	160.6	295.2	5.1	1.2	2.0	0.8	78.6	192.7	5.8	52.9	106.3	15.9	2.1	5.9	0.9
	Eri	180.1	1352	2.4	802.9	1476	25.4	5.8	10.1	3.9	393.2	963.5	29.2	264.5	531.3	79.6	10.6	29.6	4.3
Cu	CF	3.9	19.8	0.5				2.0	4.5	1.2	8.1	17.5	2.0	9.5	13.6	4.6	1.9	10.0	0.8
	Eri	19.6	99.1	2.6				10.2	22.5	6.0	40.6	87.7	9.8	47.5	67.9	23.0	9.3	50.0	3.8
Hg	CF	567.7	2292	210.8															
	Eri	22,700	91,690	8430															
As	CF	0.9	1.2	0.4															
	Eri	8.5	12.0	4.2															
Sb	CF	1.6	11.6	0.1															
	Eri	16.3	115.7	1.4															
Zn	CF	0.5	1.2	0.3				0.8	1.2	0.4	0.9	1.2	0.6						
	Eri	0.5	1.2	0.3				0.8	1.2	0.4	0.9	1.2	0.6						
Ni	CF							1.2	2.1	0.7	1.3	1.4	1.0						
	Eri							6.1	10.5	3.5	6.4	7.2	5.2						
Cr	CF										1.5	1.6	1.3						
	Eri										2.9	3.2	2.6						
Co	CF							0.8	1.6	0.4									
	Eri							3.9	7.8	2.0									
	CD	610.6	2597	212.6	160.6	295.2	5.1	6.0	11.4	3.5	90.4	214.5	10.8	62.4	119.8	20.5	4.0	15.9	1.6
	IR	22,930	93,270	8442	802.9	1476	25.4	26.8	52.2	15.9	443.9	1063	47.5	312.0	599.2	102.6	19.9	79.6	8.1

**Table 3:** Non-carcinogenic risks (HI) of heavy metals in shooting range soils.

Metal	Range 1			Range 2			Range 3			Range 4			Range 5-1			Range 5-2		
	Mean	Max	Min	Mean	Max	Min	Mean	Max	Min	Mean	Max	Min	Mean	Max	Min	Mean	Max	Min
Pb	3.26 × 10^−1^	2.45	4.35 × 10^−3^	1.45	2.67	4.60 × 10^−2^	1.06 × 10^−2^	1.83 × 10^−2^	7.14 × 10^−3^	7.12 × 10^−1^	1.74	5.29 × 10^−2^	4.79 × 10^−1^	9.62 × 10^−1^	1.44 × 10^−1^	1.92 × 10^−2^	5.35 × 10^−2^	7.86 × 10^−3^
Cu	2.66 × 10^−3^	1.35 × 10^−2^	3.49 × 10^−4^				1.39 × 10^−3^	3.06 × 10^−3^	8.19 × 10^−4^	5.51 × 10^−3^	1.19 × 10^−2^	1.33 × 10^−3^	6.45 × 10^−3^	9.23 × 10^−3^	3.13 × 10^−3^	1.26 × 10^−3^	6.80 × 10^−3^	5.12 × 10^−4^
Hg	1.54 × 10^−1^	6.23 × 10^−1^	5.73 × 10^−2^															
As	3.81 × 10^−2^	5.35 × 10^−2^	1.87 × 10^−2^															
Sb	6.00 × 10^−3^	4.26 × 10^−2^	5.18 × 10^−4^															
Zn	1.62 × 10^−4^	3.69 × 10^−4^	8.19 × 10^−5^				2.29 × 10^−4^	3.68 × 10^−4^	1.30 × 10^−4^	2.75 × 10^−4^	3.68 × 10^−4^	1.93 × 10^−4^						
Ni							1.97 × 10^−3^	3.40 × 10^−3^	1.15 × 10^−3^	2.06 × 10^−3^	2.34 × 10^−3^	1.69 × 10^−3^						
Cr										4.26 × 10^−2^	4.71 × 10^−2^	3.86 × 10^−2^						
Co							8.21 × 10^−4^	1.65 × 10^−3^	4.21 × 10^−4^									
Sum	5.27 × 10^−1^	3.18	8.13 × 10^−2^	1.45	2.67	4.60 × 10^−2^	1.50 × 10^−2^	2.68 × 10^−2^	9.66 × 10^−3^	7.62 × 10^−1^	1.81	9.47 × 10^−2^	4.85 × 10^−1^	9.71 × 10^−1^	1.47 × 10^−1^	2.05 × 10^−2^	6.03 × 10^−2^	8.37 × 10^−3^

**Table 4:** Carcinogenic risks (TR) of heavy metals in shooting range soils.

Metal	Range 1			Range 3			Range 4		
	Mean	Max	Min	Mean	Max	Min	Mean	Max	Min
As	5.89 × 10^−6^	8.27 × 10^−6^	2.88 × 10^−6^						
Ni				1.29 × 10^−9^	2.23 × 10^−9^	7.52 × 10^−10^	1.35 × 10^−9^	1.53 × 10^−9^	1.11 × 10^−9^
Cr							1.75 × 10^−7^	1.94 × 10^−7^	1.59 × 10^−7^
Co				4.52 × 10^−9^	9.10 × 10^−9^	2.32 × 10^−9^			
Sum	5.89 × 10^−6^	8.27 × 10^−6^	2.88 × 10^−6^	5.81 × 10^−9^	1.13 × 10^−8^	3.07 × 10^−9^	1.77 × 10^−7^	1.95 × 10^−7^	1.60 × 10^−7^

## Supplementary Materials

The following are available online at https://www.mdpi.com/2305-6304/8/2/32/s1, Table S1: Background values and toxic response factors of the surveyed metals, Table S2: The degree of contamination and potential ecological risk (Hakanson 1980) [14], Table S3: Parameters and their definitions and values in Equations (5)–(7), Table S4: Health risk assessment parameters for various metals.
