# Supplementary material for: Ecological and Human Health Risks of Heavy Metals in Shooting Range Soils: A Meta Assessment from China
Source: Toxics. 2020 May 1;8(2):32. doi: 10.3390/toxics8020032 (PMC7356891; doi:10.3390/toxics8020032)
Supplement: Supplementary file 1 [file toxics-08-00032-s001.pdf]

Supplementary Materials: Ecological and Human Health Risks of Heavy Metals in Shooting Range Soils: a Meta Assessment from China

Juan Bai and Xiaofen Zhao

Table S1. Background values and toxic response factors of the surveyed metals.

Element	Hg	Cd	As	Sb	Pb	Ni	Cu	Co	Cr	Zn
Background value	0.065	0.087	11.2	1.21	26.0	26.9	22.6	12.7	61.0	74.2
Toxic response factor	40	30	10	10	5	5	5	5	2	1

Table S2. The degree of contamination and potential ecological risk [1].

CF	CD	Degree of Contamination	Eri	RI	Degree of Ecological Risk
CF < 1	CD < 8	Low	Eri < 40	RI < 150	Low
1 ≤ CF < 3	8 ≤ CD < 16	Moderate	40 ≤ Eri < 80	150 ≤ RI <300	Moderate
3 ≤ CF < 6	16 ≤ CD < 32	Considerable	80 ≤ Eri < 160	300 ≤ RI <600	Considerable
CF ≥ 6	CD ≥ 32	Very high	160 ≤ Eri < 320		High
			Eri ≥ 320	RI ≥ 600	Very high

Table S3. Parameters and their definitions and values in Equations 5–7.

Parameter (unit)	Definition	Value	References
ADD_{ing}, ADD_{inh}, ADD_{der} [mg/(kg·d)]	Average daily exposure dose via pathway of ingestion, inhalation, and dermal contact, respectively		
C_{metal} (mg/kg)	Heavy metal concentrations in soil		The present study
IngR (mg/d)	Ingestion rate	100	
InhR (m^3/d)	Inhalation rate	15.7	
PEF (m^3/kg)	Particle emission factor	1.36×10^9	
SL [mg/(cm^2·d)]	Skin adherence factor	0.07	
SA (cm^2)	Exposed skin area	5700	[2–4]
ABS	Dermal absorption factor	0.001	
TEF (d/a)	Exposure frequency	262.5	
ED (a)	Exposure duration	24	
BW (kg)	Body weight	60.6	
AT	Average time	Non-cancer: ED×365<br>Cancer: 70×365	

Table S4. Health risk assessment parameters for various metals.

Parameter	As	Cr	Co	Ni	Pb	Hg	Zn	Cu	Sb
Ingestion RfD	$3.00×10^{-4}$	$3.00×10^{-3}$	$2.00×10^{-2}$	$2.00×10^{-2}$	$3.50×10^{-3}$	$3.00×10^{-4}$	$3.00×10^{-1}$	$4.00×10^{-2}$	$4.00×10^{-4}$
Inhalation RfD	$3.01×10^{-4}$	$2.86×10^{-5}$	$5.71×10^{-6}$	$2.06×10^{-2}$	$3.52×10^{-3}$	$8.57×10^{-5}$	$3.00×10^{-1}$	$4.02×10^{-2}$	
Dermal RfD	$1.23×10^{-4}$	$6.00×10^{-5}$	$1.60×10^{-2}$	$5.40×10^{-3}$	$5.25×10^{-4}$	$2.10×10^{-5}$	$6.00×10^{-2}$	$1.20×10^{-2}$	$6.00×10^{-5}$
Ingestion SF	1.50								
Inhalation SF	15.1	42.0	9.80	0.84					
Dermal SF	3.66								

Note: RfD [mg/(kg·day)] is the homologous reference dose, representing the largest permissible hazard through everyday exposure. Cancer slope factor (SF) $[mg/(kg·day)]^{-1}$ represents the possibility of cancer development. Data are from the references [5–8].

References

1. Hakanson, L., An ecological risk index for aquatic pollution control. A sedimentological approach. *Water Res.* **1980,** 14, 975–1001.
2. Zhou, L.; Liu, G.; Shen, M.; Hu, R.; Sun, M.; Liu, Y., Characteristics and health risk assessment of heavy metals in indoor dust from different functional areas in Hefei, China. *Environ. Pollut.* **2019,** 251, 839–849.
3. Kurt-Karakus, P. B., Determination of heavy metals in indoor dust from Istanbul, Turkey: estimation of the health risk. *Environ. Int.* **2012,** 50, 47–55.
4. Duan, X. L., *Exposure Factors Handbook of Chinese Population (Adults)*. China Environmental Science Press: Beijing, **2013**.
5. Lu, X.; Zhang, X.; Li, L. Y.; Chen, H., Assessment of metals pollution and health risk in dust from nursery schools in Xi'an, China. *Environ. Res.* **2014,** 128, 27–34.
6. Li, L. Z.; Zhang, L. J.; Hu, G. C., The exposure and health risk assessment of the heavy metals in house dust from mineral areas, southwest of China. *Asian J. Ecotoxicol.* **2017,** 12, 235–242.
7. ur Rehman, I.; Ishaq, M.; Ali, L.; Khan, S.; Ahmad, I.; Din, I. U.; Ullah, H., Enrichment, spatial distribution of potential ecological and human health risk assessment via toxic metals in soil and surface water ingestion in the vicinity of Sewakht mines, district Chitral, Northern Pakistan. *Ecotoxicol. Environ. Saf.* **2018,** 154, 127–136.
8. De Miguel, E.; Izquierdo, M.; Gómez, A.; Mingot, J.; Barrio-Parra, F., Risk assessment from exposure to arsenic, antimony, and selenium in urban gardens (Madrid, Spain). *Environ. Toxicol. Chem.* **2017,** 36, 544–550.
